# High Work-Related Stress and Anxiety as a Response to COVID-19 Among Health Care Workers in South Korea: Cross-sectional Online Survey Study

**DOI:** 10.2196/25489

**Published:** 2021-10-22

**Authors:** Myung Hee Ahn, Yong-Wook Shin, Sooyeon Suh, Jeong Hye Kim, Hwa Jung Kim, Kyoung-Uk Lee, Seockhoon Chung

**Affiliations:** 1 Division of Psychiatry, Health Screening and Promotion Center Asan Medical Center Seoul Republic of Korea; 2 Department of Psychiatry Asan Medical Center University of Ulsan College of Medicine Seoul Republic of Korea; 3 Department of Psychology Sungshin Women's University Seoul Republic of Korea; 4 Department of Clinical Nursing University of Ulsan Seoul Republic of Korea; 5 Department of Clinical Epidemiology and Biostatistics Asan Medical Center University of Ulsan College of Medicine Seoul Republic of Korea; 6 Department of Psychiatry Uijeongbu St Mary’s Hospital College of Medicine, The Catholic University of Korea Seoul Republic of Korea

**Keywords:** COVID-19, health personnel, occupational stress, anxiety, depression, stress, mental health, South Korea, health care worker, assessment, intervention

## Abstract

**Background:**

The COVID-19 outbreak had a severe impact on health care workers' psychological health. It is important to establish a process for psychological assessment and intervention for health care workers during epidemics.

**Objective:**

We investigated risk factors associated with psychological impacts for each health care worker group, to help optimize psychological interventions for health care workers in countries affected by the COVID-19 pandemic.

**Methods:**

Respondents (n=1787) from 2 hospitals in Korea completed a web-based survey during the period from April 14 to 30, 2020. The web-based survey collected demographic information, psychiatric history, and responses to the 9-item Stress and Anxiety to Viral Epidemics (SAVE-9), 9-item Patient Health Questionnaire (PHQ-9), and 7-item Generalized Anxiety Disorder-7 (GAD-7) scales. We performed logistic regression to assess contributing factors as predictor variables, using health care workers’ depression as outcome variables.

**Results:**

Among 1783 health care workers, nursing professionals had significantly higher levels of depression (PHQ-9 score: meannurse 5.5, SD 4.6; meanother 3.8, SD 4.2; *P*<.001), general anxiety (GAD-7 score: meannurse 4.0, SD 4.1; meanother 2.7, SD 3.6; *P*<.001), and virus-related anxiety symptoms (SAVE-9 score: meannurse 21.6, SD 5.9; meanother 18.6, SD 6.3; *P*<.001). Among nursing professionals, single workers reported more severe depressive symptoms than married workers (PHQ-9 score ≥10; meannurse 20.3%; meanother 14.1%; *P*=.02), and junior (<40 years) workers reported more anxiety about the viral epidemic (SAVE-9 anxiety score; meannurse 15.6, SD 4.1; meanother 14.7, SD 4.4; *P*=.002). Logistic regression revealed that hospital (adjusted odds ratio [OR] 1.45, 95% CI 1.06-1.99), nursing professionals (adjusted OR 1.37, 95% CI 1.02-1.98), single workers (adjusted OR 1.51, 95% CI 1.05-2.16), higher stress and anxiety to the viral infection (high SAVE-9 score, adjusted OR 1.20, 95% CI 1.17-1.24), and past psychiatric history (adjusted OR 3.26, 95% CI 2.15-4.96) were positively associated with depression.

**Conclusions:**

Psychological support and interventions should be considered for health care workers, especially nursing professionals, those who are single, and those with high SAVE-9 scores.

## Introduction

COVID-19 is a highly contagious respiratory disease first reported in December 2019 in Wuhan, Hubei Province, China [1]. In Korea, the first patient was diagnosed on January 20, 2020, and the number of infections increased rapidly, exceeding 5000 infections within 6 weeks as people who participated in religious events were infected [2]. The Korean government raised the country’s infectious disease alert level to the highest level on February 23, 2020 [3], set up and operated 638 screening clinics to quickly examine individuals with fever or respiratory symptoms, expanded specialized infectious disease hospitals nationwide to treat patients with severe symptoms, and allocated 10,000 beds for the treatment of patients with mild symptoms. People were obligated to follow strong social distancing measures, such as voluntarily refraining from going out and restricting movement set by the Korean government for at least 2 weeks. Two months after the government raised the alert to the highest level, the average daily number of new infections gradually decreased and remained under 20 from April 18, 2020, until July 2021, when the number of confirmed infections increased again, exceeding 1000 per day. As of July 9, 2021, Korea reported a total of 165,344 individuals with confirmed infections, of whom 15,462 were quarantined (152,498 completed quarantine; 10,810 quarantined) and 2036 were deceased [3].

Health care workers on the frontlines play a major role in preventing the spread of COVID-19 by implementing the government’s strong countermeasures. Despite their heroic efforts during the early phase of the pandemic, their mental health faces a considerable threat. In other disasters, health care workers take care of patients who have been hurt, but they are not themselves affected directly by the disaster. In contrast, health care workers can be directly affected during epidemics. For health care workers who are in close contact with patients with confirmed or suspected COVID-19, lack of personal protective equipment, work overload, poor infection control, and pre-existing medical conditions were identified as risk factors for the disease [4]. Previous studies conducted during severe acute respiratory syndrome (SARS), influenza A/H1N1, and the Middle East Respiratory Syndrome (MERS) outbreaks showed that health care workers face the fear of infecting family, friends, and colleagues [5-7]; had increased workloads and reluctance to work; perceived stigmatization, coping by avoiding crowds and colleagues; and felt scrutinized [7-9]. Many experienced severe emotional stress, such as anxiety, worrying, burnout, insomnia, and depressive symptoms, and were diagnosed with acute stress disorder or posttraumatic stress disorder [6,7,10-13]. The rate of distress among health care workers is higher than that in the general population [14]. Similarly, recent studies have shown that a significant proportion of health care workers experienced psychological impacts during the COVID-19 outbreak, such as depression, anxiety, and stress [15-21]. These studies reported that the psychological impact of COVID-19 on health care workers was highly associated with their sociodemographic characteristics and was related to stress vulnerability or social support. Occupation and workplace differences are also important factors. Female health care workers, nurses, and frontline workers directly engaged in the diagnosis, treatment, and care of patients with COVID-19 are particularly vulnerable to mental health symptoms [15,20].

As of July 2021, the COVID-19 pandemic has been ongoing for more than a year and a half. Psychological problems and exhaustion are not only a burden on health care workers but could also affect society as a whole, by threatening essential health care services or resulting in severe staff shortages. It is, therefore, important to establish a process for psychological assessment and intervention for health care workers affected by epidemics. Studies have assessed psychological symptoms using well-known scales such as the 7-item Generalized Anxiety Disorder scale (GAD-7), 9-item Patient Health Questionnaire (PHQ-9) [15,16,18], 6-item version of State-Trait Anxiety Inventory, and Center for Epidemiologic Studies Depression Scale [19]. However, these scales are not specific to viral epidemics but apply to general situations. Few have used specialized rating scales for health care workers in epidemics. One such study [13], in which 150 health care workers participated, developed a questionnaire for health care workers during the MERS outbreak and the 6-month period after the outbreak ended. However, the questionnaire lacks qualitative validity and comparison with other scales, rendering it impractical for use. Therefore, a rating scale that is brief, specific to a viral epidemic, and tailored to health care workers is necessary to assess their work-related stress in response to a viral epidemic.

In this study, we aimed to assess the stress and anxiety response of health care workers specific to the COVID-19 pandemic, by using the Stress and Anxiety to Viral Epidemics-9 (SAVE-9) scale [22], which we developed to measure specific anxiety responses of health care workers to the viral epidemic, along with other well-known scales to assess general anxiety and depression. In addition, we investigated which demographic risk factors, such as type of health care job, age, sex, and marital status, affected stress, anxiety, and depression symptoms during the pandemic, and we screened health care workers who were having an anxiety response to the viral epidemic but who had been identified by preexisting rating scales (not specific to the viral epidemic) as not having general anxiety, in order to highlight the need for establishing psychosocial support services for evidence-based rapid evaluations and psychological crisis interventions for vulnerable health care workers during any future infectious disease outbreak.

## Methods

### Study Site

This study was conducted among health care workers at the Asan Medical Center, a tertiary hospital (2705 beds; 7970 health care workers) in Seoul, and the Uijeongbu St. Mary’s Hospital, a secondary hospital (716 beds; 1800 health care workers) in Uijeongbu, Gyeonggi province, South Korea. During the outbreak, due to the rapid increase in the number of confirmed COVID-19 cases in Uijeongbu St. Mary’s Hospital, the entire hospital was placed in isolation for 3 weeks starting from March 1, 2020. During cohort isolation, outpatient departments were closed, and the discharge of in-patients was withheld. Wards exposed to patients with confirmed COVID-19 were quarantined and only essential medical staff were allowed to enter the wards. Quarantined individuals were regularly tested for COVID-19, and those who tested negative remained in quarantine, whereas those who tested positive were transferred to a designated COVID-19 treatment institution. On May 11, 2020, the hospital was restored to full functionality.

A patient who had visited Uijeongbu St. Mary’s Hospital on March 25, 2020, was admitted to the emergency room of Asan Medical Center on March 26, 2020, and was confirmed to have COVID-19 on March 31, 2020. Afterward, 4 wards were placed in cohort isolation and 57 health care workers were quarantined. Cohort isolation in the wards was lifted on April 15, 2020, and Asan Medical Center the COVID-19 intensive care medical institution status was removed on April 19, 2020.

### Participants and Procedure

The survey was conducted from April 14 to 18, 2020, at Uijeongbu St. Mary’s Hospital and from April 20 to 30, 2020, at Asan Medical Center. We used a cross-sectional, anonymous survey design to assess the psychological impact on health care workers. We advertised this study through notice boards at the 2 hospitals, and 1787 health care workers responded voluntarily. To avoid face-to-face contact, respondents completed the questionnaires through a web-based survey platform. Respondents were not compensated for their participation. This study was approved by the Asan Medical Center institutional review board (2020-0580, UC20RADI0090). Written informed consent was waived, as the respondents could declare, while answering the web-based survey, whether or not they agreed to the use of their information for the study.

Health care workers were classified into 5 groups based on the International Standard Classification of Occupations 2008 revision (ISCO) [23]: medical doctors (ISCO codes: 2211 and 2212); nursing professionals (ISCO code: 2221); health associate professionals (ISCO codes: 2240, 2261, 2262, 2264, 2265, 2266, 2267, 3211, 3212, 3213, 3214, 3221, 3252, and 3253); health management and support personnel (ISCO codes: 1342, 2131, 2133, 3141, and 3344); and clerical support workers, service and sales workers, trade workers, and plant and machine operators; and health service provided not classified elsewhere.

### Assessment Measures

#### SAVE-9 scale

The SAVE-9 scale was developed to assess work-related stress and anxiety response of health care workers to the COVID-19 pandemic [22]. Respondents rated agreement with each item on a 5-point scale from 0 (never) to 4 (always). In the previous validation study [22], satisfactory internal consistency (Cronbach α=.795) was observed, and a 2-factor structure was adopted: (1) anxiety about viral epidemics and (2) work-related stress associated with viral epidemics. A SAVE-9 score of ≥22 (or total anxiety subcategory score ≥15) was comparable to at least a mild degree with GAD-7 total score. We used the Korean version of the SAVE-9 scale, since it was originally developed in the Korean language.

#### PHQ-9

PHQ-9 is a self-administered, 9-item questionnaire used to assess depression. Each item is scored on a 3-point scale from 0 (not at all) to 3 (nearly every day). Scores can range from 0 to 27, with higher scores reflecting greater symptom severity. A PHQ-9 score >10 indicates depression [24]. In this study, we used the Korean version of the PHQ-9 scale [25].

#### GAD-7

GAD-7 is a self-administered, 7-item questionnaire specific to general anxiety. Each item is scored on a 3-point scale from 0 (not at all) to 3 (nearly every day). Scores can range from 0 to 21, with higher scores reflecting greater symptom severity. In this study, a score ≥5 was used for mild anxiety [26], as we wished to screen health care workers with at least mild degrees of anxiety. In this study, we used the Korean version of the GAD-7 scale [25].

#### Sociodemographic data

Sex, age, marital status, type of health care job, and years of employment were collected. Additionally, respondents were asked whether they had a current or previous diagnosis of depression, anxiety, or insomnia.

### Analysis

Statistical analyses were performed using SPSS software (version 21.0 for Windows; IBM Corp). The clinical characteristics were summarized as mean (SD) values. To calculate frequency, the number of each sample was divided by the total number of samples in each health care worker group. The student *t* test (2-tailed) was used for continuous variables, and the chi-square test (2-tailed) was used for categorical variables for between-group analyses. The level of significance for all analyses was *P*<.01. Logistic regression analysis was conducted to explore risk factors for health care worker depression. Finally, the additional value (ie, detection of those who were not screened through GAD-7) of the SAVE-9 was estimated using the McNemar test. To obtain robust odds ratios (OR), considering previously (or clinically) important factors, variables with *P*<.10 in univariate analysis were included.

## Results

A total of 1023 Asan Medical Center health care workers and 764 Uijeongbu St. Mary’s Hospital health care workers participated in the web-based survey. We analyzed data from 1783 health care workers (Table 1) after excluding 4 responses of health care workers who did not agree to the use of their responses in this study. Of 1783 respondents, 76.1% (1356) were female, 52.7% (939) were single. The proportion of participants was high among those in their 20s and 30s. Asan Medical Center had more nursing professionals as respondents, more health care workers with psychiatric histories, and higher PHQ-9, GAD-7, and SAVE-9 work-related stress subcategory scores than Uijeongbu St. Mary’s Hospital.

Among the 5 categories of health care workers, nursing professionals were younger (75.1% of juniors in nursing professionals, 60.2% in all workers excluding nursing professionals, *P*<.001), more depressed (PHQ-9 score: 5.5, SD 4.7, vs 3.8, SD 4.2; *P*<.001), and more anxious (GAD-7 score: 4.0, SD 4.1, vs 2.7, SD 3.6; *P*<.001) than workers in all other groups (Tables 2 and 3). The SAVE-9 scale score was significantly correlated with PHQ-9 score for all health care worker groups (all *P*<.001). In nursing professionals, single workers reported more depressive symptoms (higher proportion of workers whose PHQ-9 score ≥10) compared with married workers (*P*=.008). Excluding nursing professionals, other groups’ PHQ-9 scores did not differ significantly with respect to sex, age, or marital status; however, female health care workers reported higher anxiety (higher proportions of GAD-7 score ≥5) than male health care workers (*P<.*001), and married health care workers reported more anxiety than single health care workers (*P*=.010). Especially among all married health care workers, nursing professionals had significantly higher SAVE-9 (21.3, SD 5.7, vs 19.3, SD 6.1; *P*<.001), GAD-7 (3.9, SD 3.8, vs 2.9, SD 3.5; *P*<.001), and PHQ-9 scores (4.9, SD 4.5, vs 3.9, SD 4.1; *P*<.001) than those of other health care workers. In nursing professionals, junior workers (<40 years) were more anxious about the viral epidemic situation (*P*=.002); junior (*P*<.001) and single workers (*P*=.001) were more stressed about their work. Female workers among all workers, excluding nursing professionals, were more anxious about the viral epidemic (*P*<.001) and felt more stressed (*P*<.001).

**Table 1:** Demographic characteristics of the respondents.

Variables		ASAN medical center (n=1019), n (%)	Uijeongbu St. Mary’s Hospital (n=764), n (%)	*P* value	All (n=1783), n (%)
**Gender**				<.001	
	Male	211 (20.7)	216 (28.3)		427 (23.9)
	Female	808 (79.3)	548 (71.7)		1356 (76.1)
**Age**				<.001	
	20-29 years	309 (30.3)	287 (38.5)		596 (33.4)
	30-39 years	387 (38.0)	222 (29.8)		609 (34.2)
	40-49 years	253 (24.8)	161 (21.6)		414 (23.2)
	50-59 years	70 (6.9)	74 (9.9)		144 (8.1)
	60-65 years	0 (0.0)	1 (0.1)		1 (0.1)
**Marital status**				.304	
	Single	529 (52.3)	410 (53.7)		939 (52.7)
	Married	482 (47.7)	354 (46.3)		836 (46.9)
**Categories of health care workers**				<.001	
	Medical doctors	192 (18.8)	100 (13.1)		292 (16.4)
	Nursing professionals	596 (58.7)	369 (48.3)		967 (54.2)
	Health associate professionals	126 (12.4)	120 (15.7)		246 (13.8)
	Health management and support personnel	83 (8.1)	85 (11.1)		168 (9.4)
	Health service provided not elsewhere classified	20 (2.0)	90 (11.8)		110 (6.2)
Past psychiatric history (yes)		129 (12.7)	49 (6.4)	<.001	178 (10.0)
Years of employment (year)		9.9 (9.0)	9.5 (9.3)	.369	9.7 (9.1)
**Assessment measures**					
	Patient Health Questionnaire–9	4.9 (4.6)	4.4 (4.4)	.006	4.7 (4.5)
	Generalized Anxiety Disorder–7	3.7 (4.0)	3.0 (3.7)	<.001	3.4 (3.9)
	SAVE-9^a^	20.3 (5.7)	20.2 (7.0)	.642	20.3 (6.3)
	Anxiety subcategory of SAVE-9	14.2 (4.2)	14.7 (4.9)	.046	14.4 (4.5)
	Work-related stress subcategory of SAVE-9	6.1 (2.3)	5.5 (2.7)	<.001	5.8 (2.5)

^a^SAVE-9: Stress and Anxiety to Viral Epidemics-9.

**Table 2:** Clinical characteristics of respondents by health care worker category.

Variables		Nursing professionals (n=967)	Medical doctors (n=292)	Health associate professionals (n=246)	Health management and support personnel (n=168)	Health service provided not elsewhere classified (n=110)	All workers excluding nursing professionals (n=816)
**Age**							
	Junior	718 (75.1)	215 (73.9)	152 (62.3)	77 (46.7)	43 (39.8)	487 (60.2)
	Senior	238 (24.9)	76 (26.1)	92 (37.7)^a^	88 (53.4)^a^	65 (60.2)^a^	321 (31.8)^a^
Sex (female)		933 (96.5)	122 (41.8)^a^	114 (46.3)^a^	103 (61.3)^a^	84 (76.4)^a^	423 (51.8)^a^
Past psychiatric history		90 (9.3)	33 (11.3)	18 (7.3)	27 (16.2)^b^	10 (9.2)	88 (10.8)
Marital status (married)		396 (41.1)	140 (48.1)^c^	143 (58.6)^a^	94 (56.3)^a^	63 (57.3)^d^	440 (54.2)^a^
Years of employment		10.1 (8.6)	6.6 (7.4)^a^	10.3 (11.0)	11.5 (10.4)	10.4 (9.4)	9.2 (9.7)

^a^*P*<.001 compared to the nursing professionals group.

^b^*P*=.007 compared to the nursing professionals group.

^c^*P*=.035 compared to the nursing professionals group.

^d^*P*=.001 compared to the nursing professionals group.

**Table 3:** Clinical symptom assessment of the participants by category of health care worker (n=1783)

				Nursing professionals (n=967)		Medical doctors (n=292)		Health associate professionals (n=246)		Health management and support personnel (n=168)		Health service provided not elsewhere classified (n=110)		All workers excluding nursing professionals (n=816)
**PHQ-9^a^ score**				5.5 (4.6)		2.9 (3.4)^b^		3.8 (4.2)^b^		4.6 (4.7)		4.4 (4.5)		3.8 (4.2)^b^
	**Score ≥10, n (%)**													
		Junior	138 (19.2)		11 (5.1)		15 (9.8)		11 (14.3)		5 (11.6)		42 (8.6)	
		Senior	33 (13.9)		4 (5.3)		10 (10.9)		8 (9.1)		9 (13.8)		31 (9.7)	
	**Score ≥10, n (%)**													
		Male	4 (11.8)		9 (5.3)		9 (6.8)		9 (13.8)		0 (0.0)		27 (6.9)	
		Female	168 (18.0)		6 (4.9)		16 (14.0)		10 (9.7)		14 (16.7)^c^		46 (10.9)	
	**Score ≥10, n (%)**													
		Married	56 (14.1)		7 (5.0)		14 (9.8)		9 (9.6)		9 (14.3)		39 (8.9)	
		Single	115 (20.3)^d^		8 (5.3)		10 (9.9)		10 (13.7)		5 (10.6)		33 (8.9)	
**GAD-7^e^ score**				4.0 (4.1)		2.0 (3.0)^b^		3.0 (3.9)^f^		3.4 (4.0)		2.7 (3.1)^g^		2.7 (3.6)^b^
	**Score ≥5, n (%)**													
		Junior	257 (35.8)		34 (16.3)		37 (24.3)		30 (39.0)		8 (18.6)		109 (22.7)	
		Senior	85 (35.7)		11 (14.5)		23 (25.0)		26 (30.2)		18 (27.7)		78 (24.5)	
	**Score ≥5, n (%)**													
		Male	12 (35.3)		23 (14.1)		25 (18.9)		18 (27.7)		1 (3.8)		67 (17.4)	
		Female	333 (35.7)		22 (18.0)		36 (31.6)		39 (38.6)		25 (29.8)^h^		122 (29.0)^h^	
	**Score ≥5, n (%)**													
		Married	142 (35.9)		27 (19.3)		39 (27.3)		33 (35.1)		17 (27.0)		116 (26.4)	
		Single	202 (35.6)		18 (12.5)		22 (21.8)		24 (33.8)		9 (19.1)		73 (20.1)^h^	
SAVE-9^i^ score				21.6 (5.9)		17.2 (6.1)^b^		20.2 (5.9)^b^		18.9 (6.3)^b^		18.2 (6.8)^b^		18.6 (6.3)^b^
**SAVE-9 anxiety score**				15.4 (4.2)		12.0 (4.6)^b^		14.7 (4.3)		13.4 (4.5)^b^		13.3 (4.8)^b^		13.3 (4.6)^b^
	**Age**													
		Junior	15.6 (4.1)		11.6 (4.7)		14.8 (4.1)		13.8 (4.9)		13.6 (5.2)		13.1 (4.8)	
		Senior	14.7 (4.4)^j^		13.0 (4.3)		14.5 (4.7)		13.1 (4.0)		12.9 (4.6)		13.5 (4.5)	
	**Gender**													
		Male	14.1 (5.8)		11.3 (4.6)		14.2 (4.4)		12.8 (4.8)		11.1 (3.9)		12.5 (4.7)	
		Female	15.4 (4.1)		12.9 (4.4)^k^		15.3 (4.1)		13.7 (4.2)		13.9 (4.9)^l^		13.9 (4.5)^h^	
	**Marital status**													
		Married	15.4 (4.0)		12.8 (4.4)		14.6 (4.6)		13.7 (4.2)		13.9 (4.5)		13.7 (4.5)	
		Single	15.4 (4.3)		11.3 (4.6)		14.9 (3.9)		13.0 (4.7)		12.3 (5.2)		12.7 (4.7)^h^	
**SAVE-9 work-related stress score**				6.3 (2.5)		5.3 (2.2)^b^		5.5 (2.4)^b^		5.5 (2.4)^m^		5.0 (2.6)^b^		5.4 (2.4)^b^
	**Age**													
		Junior	6.4 (2.5)		5.4 (2.2)		5.5 (2.4)		5.7 (2.6)		5.0 (2.7)		5.4 (2.4)	
		Senior	5.7 (2.4)^h^		5.0 (2.3)		5.5 (2.5)		5.4 (2.3)		4.9 (2.6)		5.2 (2.4)	
	**Gender**													
		Male	5.8 (3.0)		4.9 (2.4)		5.2 (2.3)		5.1 (2.7)		3.5 (2.0)		4.9 (2.4)	
		Female	6.3 (2.5)		5.8 (1.9)^h^		5.9 (2.5)		5.8 (2.2)		5.4 (2.7) ^h^		5.8 (2.3)^h^	
	**Marital status**													
		Married	5.9 (2.4)		5.3 (2.2)		5.8 (2.4)		5.5 (2.7)		5.4 (2.7)		5.5 (2.4)	
		Single	6.5 (2.6)^h^		5.3 (2.3)		5.2 (2.4)		4.3 (2.4)		4.2 (2.4)		5.2 (2.4)	

^a^PHQ-9: Patient Health Questionnaire–9.

^b^*P*<.001 compared to nursing professionals group.

^c^*P*=.026 among each health care worker group.

^d^*P*=.014 among each health care worker group.

^e^GAD-7: Generalized Anxiety Disorder-7.

^f^*P*=.017 compared to nursing professionals group.

^g^*P*=.031 compared to nursing professionals group.

^h^*P*<.001 among each health care worker group.

^i^SAVE-9: Stress and Anxiety to Viral Epidemics-9.

^j^*P*=.002 among each health care worker group.

^k^*P*=.00 among each health care worker group3.

^l^*P*=.008 among each health care worker group.

^m^*P*=.030 compared to nursing professionals group.

**Figure 1:**
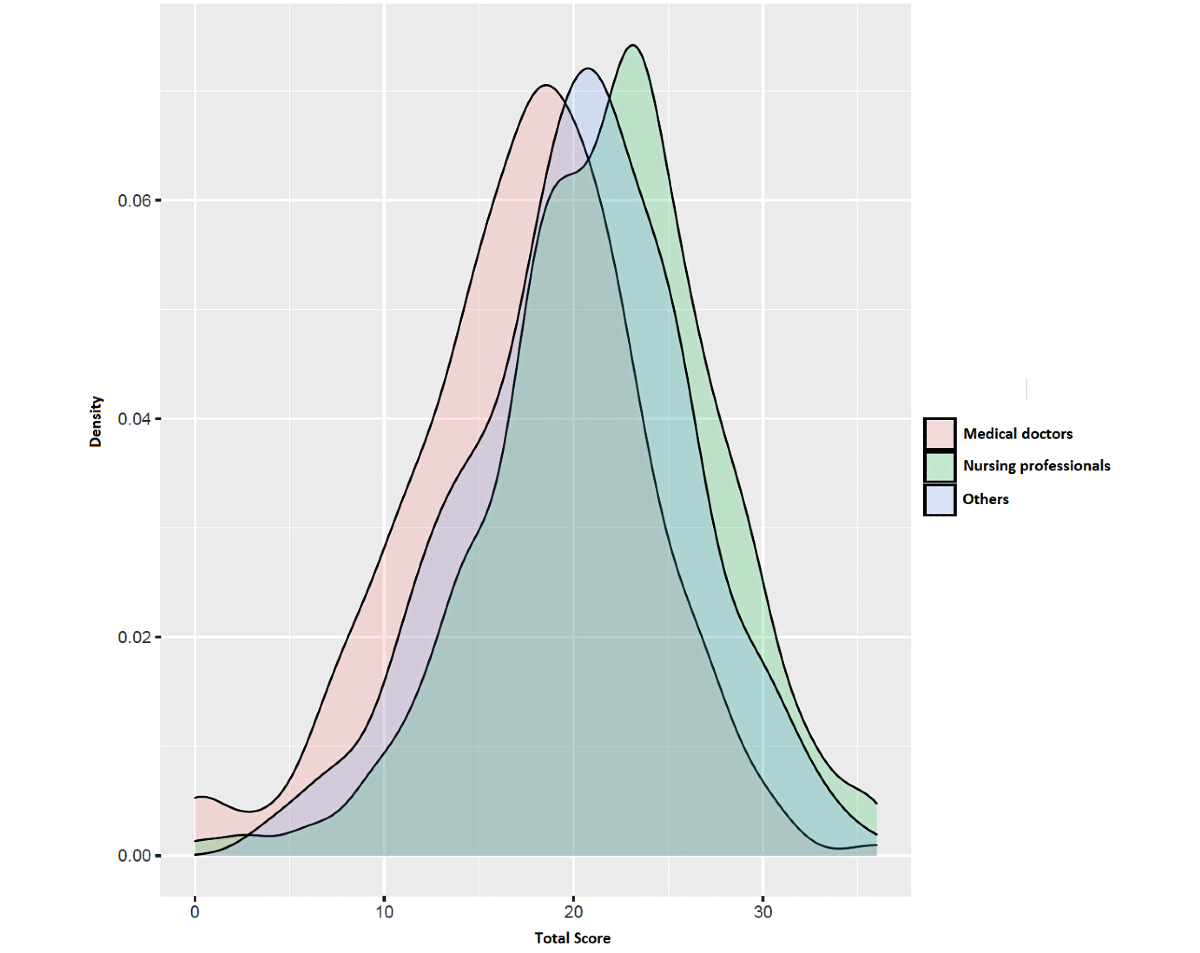
Stress and Anxiety to Viral Epidemics–9 score distributions for health care worker groups.

Compared with those of medical doctors and other groups, nursing professionals SAVE-9 scores were higher (Figure 1).

Hospital (Asan Medical Center: adjusted OR 1.45, 95% CI 1.06-1.99), nursing professionals (adjusted OR 1.37, 95% CI 1.02-1.98), single workers (adjusted OR 1.51, 95% CI 1.05-2.16), higher stress and anxiety to the viral infection (high SAVE-9 score: adjusted OR 1.20, 95% CI 1.17-1.24), and past psychiatric history (adjusted OR 3.26, 95% CI 2.15-4.96) were positively associated with depression (Table 4).

Among respondents, 534 (29.9%) health care workers were classified as having high anxiety using the GAD-7 total score (GAD-7 score >5). Among health care workers who were classified as not having high anxiety (n=1240), 400 (22.4%) health care workers were newly screened as having stress and anxiety due to the viral epidemic based on SAVE-9 scores (κ=0.351, *P*<.001).

**Table 4:** Logistic regression analysis to explore predictor variables for depression.

Explanatory variables	Crude OR^a^ (95% CI)	*P* value	Adjusted OR (95% CI)	*P* value
Asan Medical Center (vs Uijeongbu St. Mary’s Hospital)	1.43 (1.08-1.89)	.013	1.45 (1.06-1.99)	.021
Junior (vs senior)	1.36 (1.01-1.84)	.049	1.20 (0.81-1.79)	.363
Female (vs male)	2.39 (1.62-3.55)	<.001	1.11 (0.68-1.80)	.684
Single (vs married)	1.46 (1.11-1.92)	.007	1.51 (1.05-2.16)	.025
Nursing professionals (vs others)	2.20 (1.65-2.95)	<.001	1.37 (1.02-1.98)	.041
SAVE-9^b^ score	1.19 (1.16-1.23)	<.001	1.20 (1.17-1.24)	<.001
Past psychiatric history	2.47 (1.71-3.56)	<.001	3.26 (2.15-4.96)	<.001

^a^OR: odds ratio.

^b^SAVE-9: Stress and Anxiety to Viral Epidemics–9.

## Discussion

The results demonstrated that nursing professionals were more depressed, anxious, and stressed by the viral epidemic than other health care workers during the first phase of the COVID-19 pandemic. Marital status (being single) as well as anxiety and work-related stress associated with the viral epidemic were risk factors for depression among health care workers. The mean SAVE-9 score among health care workers was 20.3 (SD 6.3). Given that our previous study [22] defined mild degree symptoms of virus-related stress and anxiety as a SAVE-9 score greater than 22, this study showed similar results to those of previous studies [15,20,27,28] that showed that a high proportion of health care workers experience psychological impacts during the COVID-19 pandemic. To better fight the COVID-19 outbreak, all health care workers are being employed in activities related to epidemiological investigations and contact isolation. Along with existing health care workers at the infectious diseases departments, all workers have been recruited at screening clinics [3]. Nursing professionals directly provide care to patients with confirmed or suspected infections and their caregivers. The other health care workers in occupations that do not directly face the patients measure the temperature and sanitize the hands of all incoming people at the hospital entrance, explaining that hospital access and medical treatment are restricted to the contacts identified through epidemiological correlation. All health care workers must wear personal protective equipment at screening clinics and cohort isolation wards.

Consistent with the findings of previous studies, we found that nursing professionals were more likely to feel stress or anxiety than other health care workers [15,16,20,29,30]. Nursing professionals are in crisis as they care for patients with infections, experience fear of infectious diseases, insufficient isolation-patient-care-systems, and ethical dilemmas [31]. In addition, nurses may experience risk in situations where it is difficult to remove and re-wear a gown in-between treatments due to lack of time. Furthermore, they may face distressing situations where uncooperative patients may be exposed to direct infection [32]. As nurses interact most closely with patients and face long durations of infection risk exposure, they reported experiencing physical and emotional difficulties [32]. Feeling burdened by work changes and reacting sensitively to lack of resources may have influenced the high level of stress that nurses report experiencing. Therefore, it has been suggested that mental health and stress management programs are needed for nurses who take care of infected patients [32,33]. Through continuous infection prevention training and protective equipment training, nurses’ abilities to cope with crises and ethical dilemmas must be improved [34].

Compared with health care workers who were single, all married workers, excluding nursing professionals, scored higher on GAD-7. Owing to high medical knowledge regarding the high infectivity of the virus and the relatively insufficient medical supplies at the beginning, health care workers had high safety concerns. Married workers may worry not only about their own protection but also about the safety of their family members, including children. This finding is consistent with those of previous studies that noted that the concern for the health of oneself and one’s family was significantly higher among married workers [6,35].

However, among nursing professionals, there was no difference in GAD-7 scores of ≥5 according to marital status (single: 35.6%, married: 35.9%), compared to 26.4% of married workers and 20.1% of single workers in all other health care worker groups. Nursing professionals had higher overall depression, anxiety, and virus-related stress and anxiety than other health care workers. The Korean government’s emphasis on social distancing made it necessary for participants to submit daily results of viral symptoms monitoring and to be only at home or the hospital. Living as health care workers may have exerted a lot of pressure on them socially to improve the COVID-19 situation. Among single workers, this semicompulsory sequestration was compelled, and they experienced a greater change in life than married workers. As they could not perform daily activities to reduce their stress, their perceived negative emotions increased, and positive emotions remained relatively low [13]. In such unforeseen situations, family support is important to motivate people to continue working [13,36]. Married workers can connect more closely with their families, share things beyond work, and vent emotions better [37]. This finding indicates that single nursing professionals may need more psychological support. We also found that the hospital factor was significantly associated with depression. Asan Medical Center is one of the biggest hospitals in Korea, and there are many more patients with severe illnesses compared with those at other hospitals [38]. Compared to Uijeongbu St. Mary’s Hospital, Asan Medical Center had a higher proportion of nursing professionals, more health care workers with psychiatric histories, and higher SAVE-9 scores; these factors may have influenced the association of hospital factors with depression.

In this study, we measured anxiety symptoms among health care workers by using the SAVE-9 scale, which is used for assessing anxiety measures specific to the viral epidemic, and GAD-7 scale, which is used for measuring nonspecific anxiety. In previous SARS and MERS outbreaks, health care workers were exposed to protracted epidemics, and the unfavorable conditions resulted in a high prevalence rate of burnout and depression [39]. The COVID-19 pandemic has been ongoing for more than a year; thus, its impact on the long-term mental health of health care workers should be considered carefully. Studies [15,40] have reported the severe psychological impacts on health care workers during various phases of COVID-19; however, the rating scales used were not specific to the viral epidemic, and therefore, results did not reflect psychological stress specifically in relation to the viral epidemic. We developed SAVE-9 [22] to assess anxiety and stress of health care workers specifically in response to the COVID-19. During a pandemic, a larger number of health care workers need attention and care for maintaining essential care services. We expect that the SAVE-9 scale can be a useful tool for measuring work-related stress and anxiety response of health care workers specifically to the viral epidemic. We could identified an additional 400 (400/1783, 22.4%) health care workers as having significant stress and anxiety response to the viral epidemic to the 534 (29.9%) workers who were classified as having high anxiety using the GAD-7 scale. The GAD-7 is widely used to assess participants’ generalized anxiety, but it does not reflect the psychological stress specifically in response to viral epidemics. The viral epidemic-specific rating scale can assess the psychological state of health care workers that is specific to a situation such as the COVID-19 pandemic.

This study has some limitations. First, this was a cross-sectional study; therefore, we can suggest only associations between mental problems and COVID-19 in health care workers but not causal relationships or underlying mechanisms. Second, the survey was conducted only in 1 hospital in Seoul and 1 in Uijeongbu. Thus, the sample may have been biased. In addition, the responses might be biased, as this study utilized a self-report web-based questionnaire. Nevertheless, as the job type distribution of the sample mirrored that of the health care workers at study sites, it can be considered as substantially representative in these hospitals. Third, the questionnaire was conducted in mid-April 2020, immediately after the end of the cohort isolation. The psychological status of health care workers at the onset or peak of Korea’s COVID-19 crisis was, therefore, not assessed. Future research should focus on specific groups, incorporating according to the stage of the epidemics. We will have to collect more comprehensive data on the psychological status of health care workers in other infectious disease outbreaks. Fourth, we were unable to classify workers as parent-facing, contact, frontline health care workers, or those with a history of COVID-19 positivity or quarantine. Lai et al [15] revealed that being directly engaged in clinical activities was an independent risk factor of psychiatric symptoms. Workers with COVID-19 exposure or positivity had a 2 to 4-fold increased risk of being anxious and depressed compared with controls [41]. Moreover, quarantine activity has been shown to adversely affect mental health both during and after quarantine. Finally, the coarse categorization of health care roles may lead to biased findings. Since one of the objectives in this study was to explore which types of workers suffered the severest stress in this pandemic, we categorized them into 2 (nursing professionals vs other health care workers) groups in some of the analyses.

Despite these limitations, our study indicates that all health care workers were at psychological risk of COVID-19 and that they worried about health problems for themselves, their family, and their colleagues. Especially nursing professionals, who are the major health care workers in the medical system and work at the frontline of patient care, can easily be depressed and frustrated. In addition, their marital status (being single), past psychiatric history, and higher level of anxiety specifically in response to the viral epidemic also influence their depressive symptoms. We were able to measure anxiety response and work-related stress among health care workers during this pandemic using SAVE-9, which focuses on viral epidemic–related stress and anxiety.

## Abbreviations

GAD-7: Generalized Anxiety Disorder-7
ISCO: International Standard Classification of Occupations
MER: Middle East respiratory syndrome
PHQ-9: Patient Health Questionnaire-9
SAVE-9: Stress and Anxiety to Viral Epidemics-9
SARS: severe acute respiratory syndrome
